# Radiomics Is Effective for Distinguishing Coronavirus Disease 2019 Pneumonia From Influenza Virus Pneumonia

**DOI:** 10.3389/fpubh.2021.663965

**Published:** 2021-06-15

**Authors:** Liaoyi Lin, Jinjin Liu, Qingshan Deng, Na Li, Jingye Pan, Houzhang Sun, Shichao Quan

**Affiliations:** ^1^Department of Radiology, First Affiliated Hospital of Wenzhou Medical University, Wenzhou, China; ^2^Department of Intensive Care Unit, First Affiliated Hospital of Wenzhou Medical University, Wenzhou, China; ^3^Department of General Medicine, First Affiliated Hospital of Wenzhou Medical University, Wenzhou, China

**Keywords:** COVID-19, influenza, nomogram, radiomics, computed tomography

## Abstract

**Objectives:** To develop and validate a radiomics model for distinguishing coronavirus disease 2019 (COVID-19) pneumonia from influenza virus pneumonia.

**Materials and Methods:** A radiomics model was developed on the basis of 56 patients with COVID-19 pneumonia and 90 patients with influenza virus pneumonia in this retrospective study. Radiomics features were extracted from CT images. The radiomics features were reduced by the Max-Relevance and Min-Redundancy algorithm and the least absolute shrinkage and selection operator method. The radiomics model was built using the multivariate backward stepwise logistic regression. A nomogram of the radiomics model was established, and the decision curve showed the clinical usefulness of the radiomics nomogram.

**Results:** The radiomics features, consisting of nine selected features, were significantly different between COVID-19 pneumonia and influenza virus pneumonia in both training and validation data sets. The receiver operator characteristic curve of the radiomics model showed good discrimination in the training sample [area under the receiver operating characteristic curve (AUC), 0.909; 95% confidence interval (CI), 0.859–0.958] and in the validation sample (AUC, 0.911; 95% CI, 0.753–1.000). The nomogram was established and had good calibration. Decision curve analysis showed that the radiomics nomogram was clinically useful.

**Conclusions:** The radiomics model has good performance for distinguishing COVID-19 pneumonia from influenza virus pneumonia and may aid in the diagnosis of COVID-19 pneumonia.

## Introduction

Nowadays, the coronavirus disease 2019 (COVID-19) is a serious global health problem. COVID-19 is caused by the novel coronavirus named severe acute respiratory syndrome coronavirus 2 (SARS-COV-2), which can be transmitted through the respiratory tract and by contact and has evidence of human-to-human transmission. This episode shows the need for rapid and accurate detection and identification methods that can be used in local hospitals for the diagnosis of COVID-19. The golden diagnosis methods are the nucleic acid amplification test of the respiratory tract and the reverse transcription real-time fluorescence polymerase chain reaction of the blood specimen (1). However, the detection rate is low when the viral load is low, which may lead to false-negative results (2). The computed tomography (CT) examination has been proved to be an essential auxiliary diagnostic tool; typical CT features of COVID-19 include multifocal bilateral GGO and patchy consolidations and prominent peripherally subpleural distribution (3, 4). However, few studies focus on distinguishing COVID-19 pneumonia from other viral pneumonia.

Influenza is a significant and highly contagious disease, and the majority of viral pneumonias were caused by influenza virus types A and B in immunocompetent adults (5). Influenza A virus can cause a rapidly progressive symptom, such as acute respiratory distress syndrome, like COVID-19. The most frequent CT findings of influenza virus pneumonia were poorly defined nodules, patchy areas of peribronchial ground-glass opacity, and airspace consolidation (5, 6), which were similar to those of COVID-19 pneumonia. As we know, there are differences as well as similarities in the CT features of COVID-19 pneumonia compared with those of other viral pneumonia, and CT is still limited to differentially diagnose viral pneumonia (7).

Radiomics is a quantitative analytic method by extracting specific features from medical images (8). This novel method exhibited potential applications in assessing pulmonary nodules or masses in chest imaging study, evaluating treatment response, and predicting survival outcomes of lung cancer (9–11). Some studies demonstrated the potential of radiomics features from CT in predicting a COVID-19 patient's prognostic outcome and identification of disease severity (12, 13); however, whether CT radiomics features can be used to differentiate pneumonia caused by COVID-19 or influenza remains unclear.

We hypothesized that radiomics might provide a non-invasive method for distinguishing COVID-19 pneumonia from influenza virus pneumonia and could assist radiologists in performing an exact diagnosis. Hence, the main objective of this study was to develop and validate a radiomics model for predicting COVID-19 pneumonia in order to help clinicians in quickly and accurately eliminating the influenza virus pneumonia.

## Materials and Methods

### Patients and Ethical Approval

This study was approved by our institutional review board, and written informed consent was waived. The patients' data were collected from the First Affiliated Hospital of Wenzhou Medical University. During the period between January 25, 2020 and March 10, 2020, COVID-19 patients were included if they met the following criteria: (1) exhibiting positive results of SARS-COV-2 nucleic acids and (2) having clear chest CT scan data and with pneumonia lesion during the initial diagnosis. The influenza virus pneumonia (type A or B) patients were confirmed by nucleic acid test from February 20, 2018 to February 9, 2020. Also, they all have clear chest CT scan data during the initial diagnosis. Those with other obvious lung abnormalities (such as lung cancer, tuberculosis, silicosis, severe emphysema) or had pneumonia caused by other common bacterial or viral pathogens were excluded. Baseline clinical features were derived from medical records, including gender, age, and the symptom. COVID-19 is clinically divided into four types: mild, common, severe, and critically severe (13).

### Scanning

CT was performed for all patients using Phillips Brilliance 16, Siemens Somatom Scope 16, or GE LightSpeed VCT 64 with the following parameters: tube voltage of 120 kV, tube current-exposure time product of 50–150 mA s, scan thickness of 5 mm, and reconstruction thickness of 1.25–3 mm. The scanning range at least covered the area from the level of the apex of the lung to the costophrenic angle.

### Region-of-Interest Segmentation and Feature Extraction

The radiomics workflow began with image segmentation and feature extraction. All images were manually segmented on the AVIEW software package (Coreline Soft Co., Ltd., Korea, version 1.0.34.26). In consideration of the fact that pneumonia may have a wide range of lesions in the lung, the largest pneumonia lesion area was used in the analysis; the largest pneumonia lesion area was defined as having the largest range of lesions in a transverse section on one side of the lung, and consolidation took precedence over ground-glass opacity. The pneumonia always had larger lesion, and the lesion boundary is not clear. It is not appropriate to search a boundary for demarcation by manual operation; it may not actually get very accurate results. Therefore, we chose the lung contour to draw a cutting line that is clearer than the pneumonia lesion boundary. On the two-dimensional image, an experienced radiologist who was blind to the actual viral pneumonia results selected the transverse section of the largest pneumonia lesion area on one side of the lung and then manually delineated the region of interest (ROI) of the lung images using the system's own tools. The ROI included the transverse section of the unilateral lung tissue, which demarcated along the lung contour margin. Another radiologist checked the segmentation. Any difference in opinions was resolved through negotiation. The software automatically recognized and extracted the radiomics features. A total of 131 features were extracted, including fractal features, shape features, and texture features. The radiomics feature parameters are presented in Supplementary Data Sheet 1. After feature extraction, all the patients' image data were randomly divided into training and validation sets with a ratio of 9:1.

### Feature Selection and Predictive Model Construction

Feature selection was performed in two steps. First, for radiomics features, the Max-Relevance and Min-Redundancy (mRMR) algorithm was performed to eliminate the redundant and irrelevant features. The corresponding features were ranked according to their relevance-redundancy indexes, and 10 features in the top were retained. Second, the least absolute shrinkage and selection operator (LASSO) was conducted to further choose the optimized features for improving the final model accuracy. To demonstrate the association between the selected features and the actual COVID-19 pneumonia, we constructed a radiomics score (Radscore) as the radiomics model in the training group using multivariate backward stepwise logistic regression. The aim of multivariate logistic regression was to derive the best-fitting and most parsimonious (smallest or most efficient) model to describe the relationship between an outcome and a set of predictors. The outcome variable (dependent variables) is dichotomous (e.g., COVID-19 or not COVID-19). The independent variables are called covariates. In multivariate logistic regression, the predictor variables may be of any data level, such as categorical, ordinal, or continuous data. Put the independent and dependent variables into the calculation formula to examine a series of predictor variables and determine those that best predict COVID-19. In our study, the most predictive features were chosen, and the corresponding coefficients were evaluated. The radiomics score was calculated by summing the selected features weighted by their coefficients to reflect the COVID-19 pneumonia probability. The discriminative capability was measured using receiver operating characteristic (ROC) curve analysis. The performance of the radiomics model was evaluated in both the training set and the validation set. The area under the curve (AUC), accuracy, sensitivity, and specificity were obtained. To avoid prediction errors, we further tested the proposed model using a 1,000-iteration bootstrap analysis in both the training and validation groups.

### Development and Validation of Nomogram

The nomogram was based on a multivariable logistic regression analysis using multiple medical indicators or biological attributes and then using line segments with high or low scores for the purpose of predicting clinical outcomes or the probability of an event. In our study, the nomogram was generated according to the proposed radiomics model using the R software. The calibration of the nomogram was detected by using the calibration curves accompanied by the Hosmer–Lemeshow test (14). The Hosmer–Lemeshow test accessed the goodness-of-fit of the nomogram models, and the calibration curves measured the consistency between the predicted COVID-19 pneumonia probability and the actual COVID-19 pneumonia probability. The clinical utility of the models was measured by the decision curve analysis. Decision curves are a useful tool to evaluate the population impact of adopting a risk prediction instrument into clinical practice. Given one risk model that predicts the probability of a binary outcome, it can display estimates of the standardized net benefit by categorizing observations as “high risk.” A larger area under the decision curve suggested a better clinical utility.

### Statistical Analysis

All statistical analyses were performed using SPSS Software (Version 24, IBM, Chicago, IL) and R software (version 3.6.1; www.R-project.org). Mann–Whitney *U*-test or independent *t*-test was used for continuous variables with abnormal distributions and normal distributions, respectively. For categorical variables, chi-square test or Fisher's exact test was used. Values of *P* < 0.05 were considered to be statistically significant.

## Results

### Clinical Characteristics

In this study, 56 patients were diagnosed with COVID-19 pneumonia and 90 patients were diagnosed with influenza virus pneumonia. There were 83 male and 63 female patients, and the mean age was 54.31 ± 16.45 years. In the COVID-19 pneumonia patients, there were 35 males and 21 females, and the main age was 55.91 ± 14.92 years. In the influenza virus pneumonia patients, there were 48 males and 42 females, and the main age was 53.31 ± 17.34 years. In 56 COVID-19 patients, 30.4% was of the common type, 60.7% was of the severe type, and 8.9% was of the critically severe type.

There were no statistically significant differences in gender (*P* = 0.277) and age (*P* = 0.355) between the two groups. The most common symptoms were fever, fever with cough, and cough. In COVID-19 pneumonia patients, only two had other symptoms such as headache or weakness, and one patient had no symptom. In the influenza virus pneumonia patients, other symptoms such as sore throat, headache, and weakness were present in only one, one, and four, respectively, and three patients had no symptom. There was statistical difference in symptoms (*P* = 0.022). Patient characteristics are shown in Table 1.

**Table 1:** Baseline characteristics of patients.

	**COVID-19 pneumonia**	**Influenza virus**	***P*-value**
	**(*n* = 56)**	**pneumonia (*n* = 90)**	
Sex			0.277
Male	35	48	
Female	21	42	
Age (year)	55.91 ± 14.91	53.31 ± 17.33	0.355
Symptoms			0.022
Fever	25	19	
Fever with cough	20	40	
Cough	8	22	
Other	3	9	

### Radiomics Analysis

**Figure 1:**
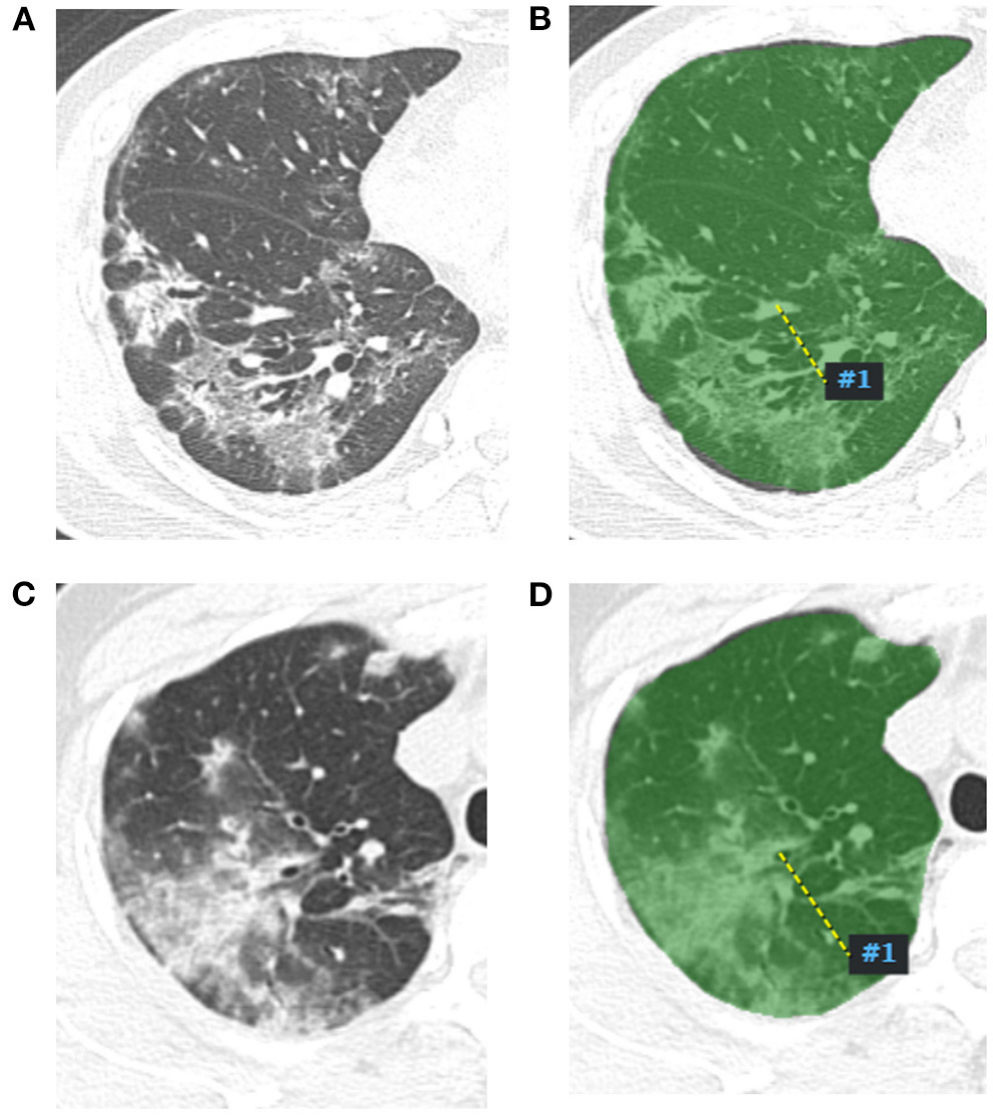
Radiomics features extract map of pneumonia lesion. A two-dimensional image of the largest pneumonia lesion area of a COVID-19 patient in the right lung **(A)**, the lesion presented as blurred patchy ground glass opacity with little consolidation. The region of interest of the largest pneumonia lesion area was manually delineated in the right lung image, which is displayed as the green area **(B)**, and artificial intelligence software automatically extracted the radiomics features and output from a computer. A two-dimensional image of the largest pneumonia lesion area of an influenza viral pneumonia patient in the right lung **(C)**, the lesion presented as ground glass opacity with some consolidation; the CT manifestation was similar with the COVID-19 pneumonia; we used the same method to extract radiomics features from the image **(D)**.

The prediction model was developed using a training set (Figure 1) that consisted of COVID-19 pneumonia and influenza virus pneumonia CT images. The mRMR was performed to get the top 10 redundant and irrelevant features, and then they were further reduced to 9 by using LASSO. Nine radiomics features were selected using mRMR and LASSO regularization methods, and these selected radiomics features were significantly associated with COVID-19 pneumonia. Finally, the coefficients of radiomics features were constructed, and the radiomics score was calculated as follows.

$$\begin{array}{l} \text{Radscore}=-1.119-0.214\;*\text{ Texture\_Histo\_Min}-0.457\\ \;*\text{ Texture\_GLCM\_MCC }+0.888\;*\text{ Shape}2\text{D\_}\\ \text{ Perimeter }-0.351\;*\text{ Texture\_GLRLM\_LRHGE}\\ \;+0.961\;*\text{ Texture\_GLCM\_DiffVariance}-0.551\\ \;*\text{ Texture\_NGTDM\_Strength}-0.553\\ \;*\text{ Texture\_GLCM\_IMC}2+0.054\;*\text{ Shape}2\text{D\_}\\ \text{ Roundness}+2.221\;*\text{ Texture\_FirstOrder\_Min} \end{array}$$

### Performance of Radiomics Model

**Figure 2:**
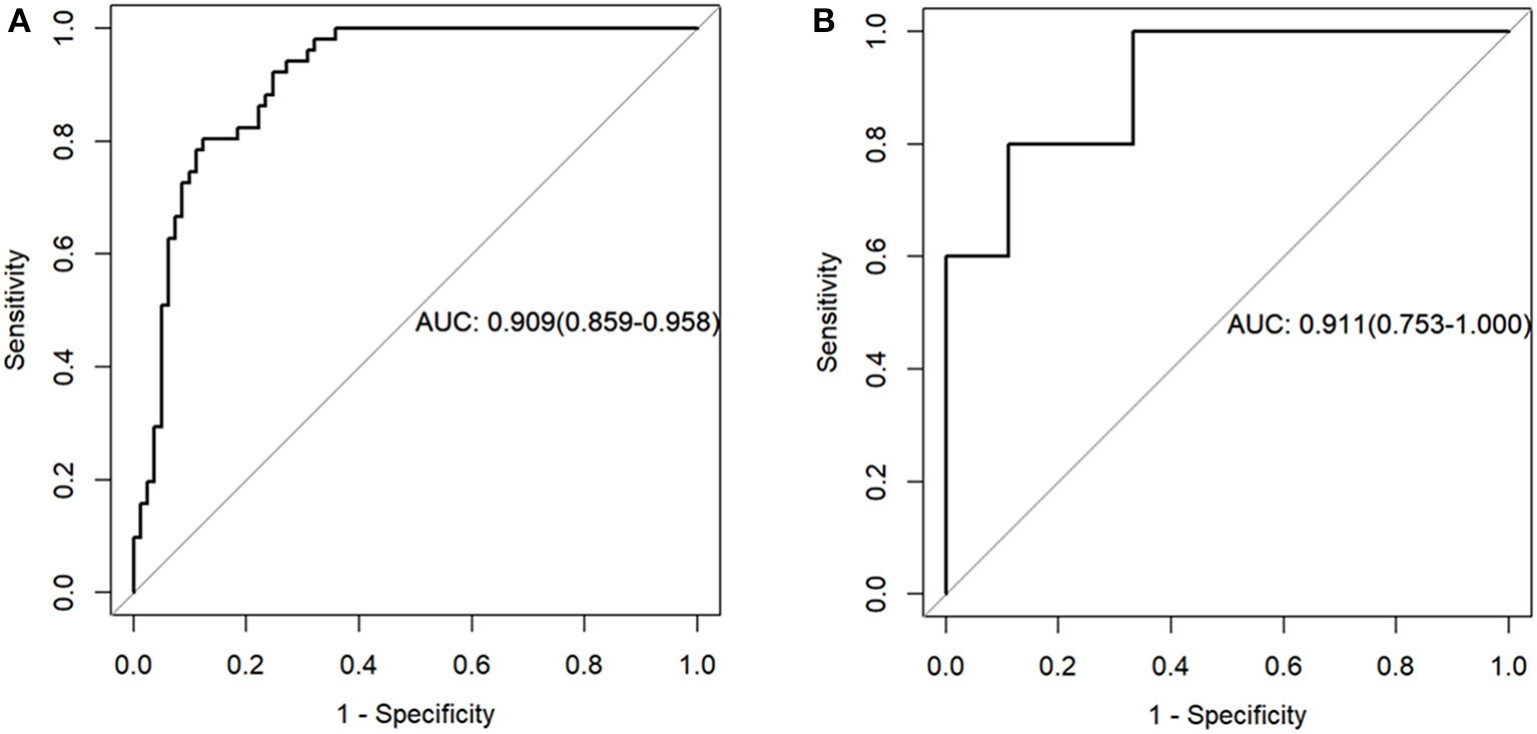
Receiver operating characteristic curves of the radiomics model in the training **(A)** and validation sets **(B)**.

The radiomics model yielded AUCs of 0.909 [95% confidence interval (CI), 0.859–0.958] and 0.911 (95% CI, 0.753–1.000) in the training and validation samples, respectively (Figure 2). These values were consistent with the AUC values calculated by using the 1,000 times bootstrap analysis in both the training and validation groups (mean ± standard deviation; the training group: 0.909 ± 0.014; the validation group: 0.910 ± 0.045). The distributions of AUCs from the bootstrap method for the radiomics model were provided as histograms in Supplementary Data Sheet 2. The accuracy, sensitivity, and specificity of the training sample were 84.8, 87.7, and 80.4%, respectively, and those of the validation sample were 85.7, 88.9, and 80.0%, respectively.

### Prediction Model

**Figure 3:**
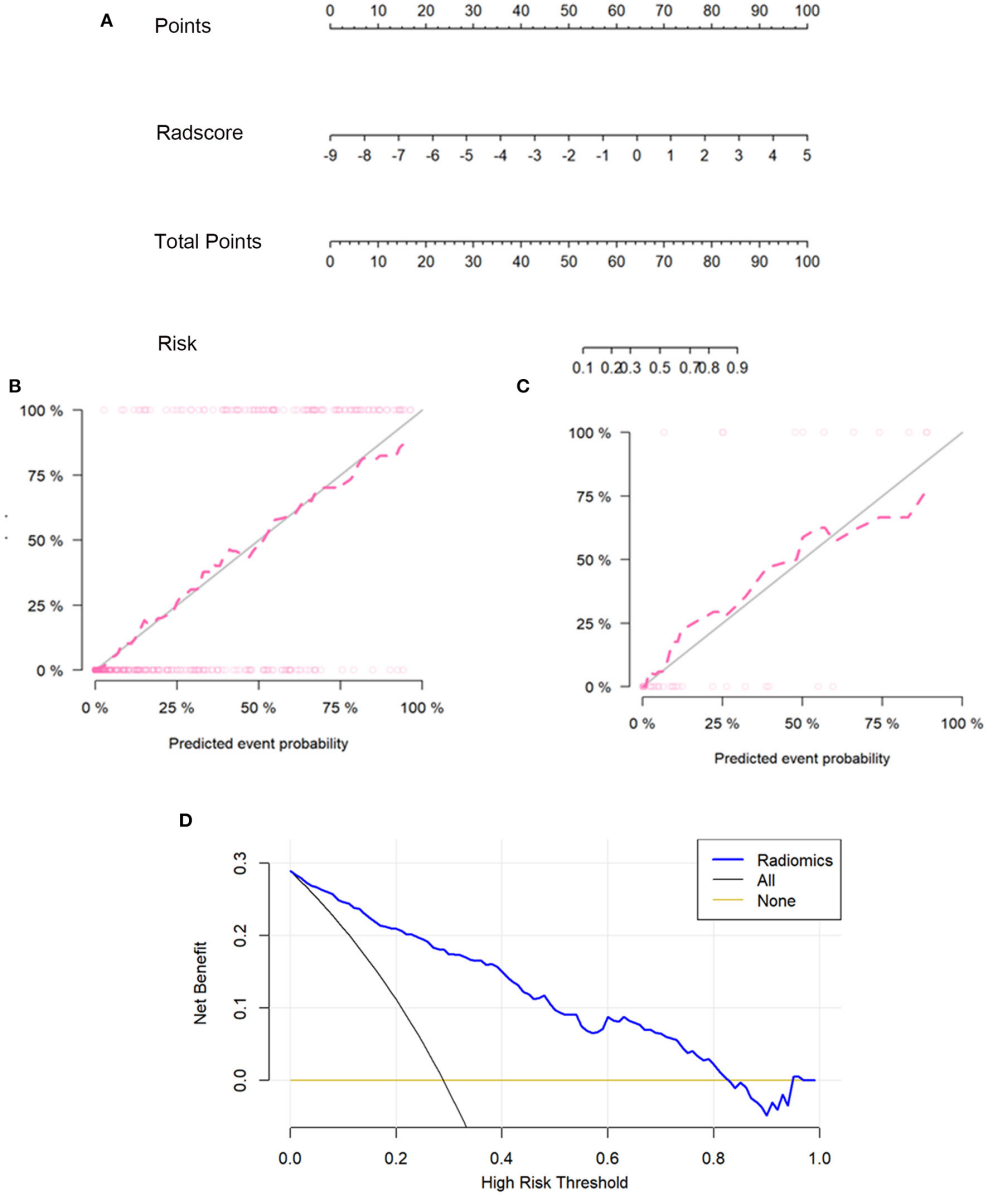
The radiomics nomogram, the calibration curves, and the decision curves. A radiomics nomogram was developed in the training set according to the radiomics score **(A)**. In the nomogram, radscore corresponded to a point due to it being a graph of a single factor; the point was the same as the total point, and the total point corresponded to the risk of COVID-19. Calibration curves of the radiomics nomogram in the training **(B)** and validation sets **(C)**. The horizontal axis of the figure was the predicted event probability, which was the probability of occurrence of the events forecasted by the prediction model, and the vertical axis was the actual event probability, which was the actual event rate of patients. The red line was the fit line and represents the actual value corresponding to the predicted value. The gray line was the reference line. The decision curve **(D)** for the radiomics score. The vertical axis was the net benefit; the horizontal axis was the high-risk threshold probability at a range of 0.0-1.0. The black line represented the hypothesis that all patients had COVID-19 pneumonia. The brown line represented the hypothesis that all patients had influenza viral pneumonia. The blue line represented the DCA of the radiomics nomogram. The decision curves showed that using the radiomics nomogram in the current study to predict COVID-19 pneumonia had more benefits.

A prediction model was developed and presented as a nomogram based on the radiomics score. It was a graph that consists of four lines: points, radscore, total points, and risk; the lines were marked off to scale and arranged in sequence. The calibration curve of the nomogram demonstrated good agreement between predicted and observed COVID-19 pneumonia in the training and validation sets. For the training group, a non-significant statistic (*P* = 0.5758) of the Hosmer–Lemeshow test suggested no significant deviation from an ideal fitting. For the validation group, the Hosmer–Lemeshow test also was a non-significant statistic (*P* = 0.3758). The calibration curve showed that the predicted curve and the actual observation curve were close, which indicated that the result was reliable. The decision curves with a larger area under the curve indicated the nomogram's clinical usefulness. The decision curve analysis showed that using the radiomics nomogram to predict COVID-19 pneumonia added more benefit than the treat-all-patients as COVID-19 pneumonia scheme or the treat-all-patients as influenza viral pneumonia scheme. All of these are shown in Figure 3.

## Discussion

In this study, we developed and validated a radiomics-based model for a non-invasive, individualized method to distinguish COVID-19 pneumonia from influenza virus pneumonia. The radiomics model demonstrated favorable discrimination in both the training set (AUC, 0.909) and the validation set (AUC, 0.911) cohorts and good calibration; DCA indicated the clinical usefulness of the radiomics model. And the influenza viral pneumonia patients more likely have fever with cough or cough than COVID-19 pneumonia patients.

Chest CT could be used to diagnosis and assess the COVID-19 patients (15). Traditional imaging methods use CT manifestations of the images for differential diagnosis. We can judge the disease by the lesion on the CT image, but the results may be subjective and inaccurate. Liu et al. found that there were differences in the CT manifestations of patients with COVID-19 and influenza. The rounded opacities and interlobular septal thickening were more commonly seen in COVID-19 compared with the influenza, and COVID-19 usually with the typical peripheral distribution (16). In a comparative study, radiologists blinded to the patient information reviewed 424 chest CT scans to differentiate COVID-19 from viral pneumonia. The accuracy of the three radiologists in differentiating COVID-19 from non-COVID-19 viral pneumonia was 83% (350 of 424), 80% (338 of 424), and 60% (255 of 424), with sensitivities of 72, 72, and 94% and specificities of 94, 88, and 24%, respectively (17). These studies showed that the CT manifestations might help us differentiate COVID-19 from influenza, but the accuracy was based on the radiologist's experience, and the results of sensitivity and specificity were not always maintained on a high level at the same time. In the clinical setting, a more comprehensive and deeper understanding of the differential diagnosis of pneumonia is needed. Radiomics is theoretically a feasible method to give rise to a deeper understanding of COVID-19 pneumonia lesions.

There is much interest in the use of radiomics for assessing COVID-19 pneumonia image data. The pixel features and spatial parameters of the image are used to quantitatively extract the pathophysiological features of the lesions that cannot be recognized by the naked eye and to reveal the special manifestations between tissues (18). Wei et al. explored the value of CT texture analysis for determining COVID-19 severity, and their prediction model of textural features showed high predictive accuracy; the Spearman correlation analysis showed that most textural features had above-moderate correlations with disease severity (13). Wu et al. aimed to develop a non-invasive and easy-to-use prognostic signature by radiomics analysis of chest CT, and they used this method to individually predict poor outcome in patients with COVID-19. Their study suggested that the chest CT radiomics signature of COVID-19 was more effective and useful, and the Radscore can successfully stratify COVID-19 patients with different survival time of poor outcome (12). Fang et al. developed a radiomics nomogram to predict COVID-19 pneumonia and help clinical decision-making, and their result showed that the radiomics model has good performance (19). In our study, the largest pneumonia lesion area was used in the analysis, since the largest lesions reflected the CT manifestations of the pneumonia lesions and had enough features to characterize the pneumonia. We only focused on COVID-19 and influenza virus pneumonia (type A or B), and we found that nine radiomics features in COVID-19 pneumonia were significantly different from those in influenza virus pneumonia. Some previous studies have used radiomics to help radiologists discriminate COVID-19 and other viral pneumonia, and the radiomics features made the image information digitalized; however, these studies mixed different types of viral pneumonia. Huang et al. included influenza A virus, influenza B virus, respiratory syncytial virus, parainfluenza virus, adenovirus, SARS coronavirus, Epstein–Barr virus, measles virus, or other viruses from nasopharyngeal swabs or bronchoalveolar lavage fluid (20). Chen et al. included influenza virus-induced, adenovirus-induced, syncytial virus-induced, and cytomegalovirus-induced pneumonias (21). Although their studies showed that the radiomics model was an effective predictive tool to distinguish COVID-19 from other viral pneumonias (with AUC 0.807–0.888), the different types of viral pneumonia had different CT findings, which is a potential confounding factor for further comparison. Our study limited the scope of viral pneumonia only for influenza virus types A and B in the comparison group. The influenza virus types A and B are among the main pathogens of community-acquired pneumonia, and they are usually required to identify with COVID-19. Our radiomics model achieved good prediction performance in both the training set (AUC, 0.909) and the validation set (AUC, 0.911). The result showed that radiomics was a valuable tool to help radiologists distinguish COVID-19 from influenza virus pneumonia. The nomogram had been widely applied to predict clinical diseases, such as the differentiation of benign and malignant cancers, cancer recurrence, and lymph node metastasis (22–24), and it was also commonly used in COVID-19 pneumonia diagnosis (20, 21). In our research, the radiomics nomogram was also developed to predict COVID-19 pneumonia and aimed to illustrate the relationship between Radscore and the risk of COVID-19 pneumonia graphically. In other words, the nomogram can quickly, intuitively, and accurately show the complex radiomics model in a graphic way, and this intuitive and convenient radiomics nomogram can be beneficial to clinical applications in distinguishing COVID-19 pneumonia from influenza viral pneumonia. Moreover, there was good calibration in both the training and validation samples for the nomogram by the Hosmer–Lemeshow test. DCA is a good tool to estimate the predicted net benefit of the model across all possible risk thresholds (25, 26). The DCA of the nomogram using the radiomics model in the current study to diagnose COVID-19 pneumonia has real utility; the treat-all-patients as COVID-19 pneumonia scheme or the treat-all-patients as influenza viral pneumonia scheme compared to the radiomics nomogram methods was significantly inferior. The calibration curve and the decision curve showed that the stability and availability of our prediction model were good; this result proved the practicability of radiomics.

Our study had several limitations. First, it was a retrospective study. Second, the radiomics features were extracted from two-dimensional images. Third, some clinical features of COVID-19 pneumonia and influenza viral pneumonia were not included to develop a model, such as the symptoms of the patients. A multicenter validation with a larger sample size is still needed in the future.

In conclusion, our study proposed a non-invasive and quantitative radiomics model for diagnosing COVID-19 based on CT imaging. Taking the ROC of the radiomics model into account, our study suggested that the CT radiomics features were more effective and ideal to distinguishing COVID-19 pneumonia from influenza viral pneumonia.

## Data Availability Statement

The raw data supporting the conclusions of this article will be made available by the authors, without undue reservation.

## Ethics Statement

The studies involving human participants were reviewed and approved by Medical ethics committee of The First Affiliated Hospital of Wenzhou Medical University. Written informed consent from the participants' legal guardian/next of kin was not required to participate in this study in accordance with the national legislation and the institutional requirements.

## Author Contributions

LL, QD, and NL: data analysis and visualization. LL and JL: writing original draft. JP, HS, and SQ: review and editing. All authors contributed to the article and approved the submitted version.

## Conflict of Interest

The authors declare that the research was conducted in the absence of any commercial or financial relationships that could be construed as a potential conflict of interest.
