# Supplementary material for: Radiomics Is Effective for Distinguishing Coronavirus Disease 2019 Pneumonia From Influenza Virus Pneumonia
Source: Front Public Health. 2021 Jun 15;9:663965. doi: 10.3389/fpubh.2021.663965 (PMC8239147; doi:10.3389/fpubh.2021.663965)
Supplement: Supplementary file 1 [file Data_Sheet_1.docx]

In this study, a number of 131 image features was extracted for each patient. These features comprised of three groups; the feature names of the image features were provided in follows. The fractal features described a small box of size 64×64×64 slides to calculate the fractal dimension of each position in the the region of interest (ROI), included “FractalFeatureParameters” and “FractalFeatures”. The shape features described the shape characteristics of the ROI, included “ShapeFeatures”. The texture features described the spatial intensity correlation and distributions of the voxels of the ROI, included “TextureFeatureParameters” and “TextureFeatures”.

| # FractalFeatureParameters |
| --- |
| BoxCountingSlide |
|  |
| # FractalFeatures |
| FractalDimension |
|  |
| # ShapeFeatures |
| Shape2D_Area(mm2) |
| Shape2D_Circularity |
| Shape2D_Compactness |
| Shape2D_Flatness |
| Shape2D_LongestAxis(mm) |
| Shape2D_OrthogonalAxis(mm) |
| Shape2D_PCAMajorStd(mm) |
| Shape2D_PCAMinorStd(mm) |
| Shape2D_Perimeter |
| Shape2D_PerimeterToAreaRatio |
| Shape2D_Roundness |
|  |
| # TextureFeatureParameters |
| GLCMBins |
| GLDMBins |
| GLDMCutoff |
| GLDMDistance |
| GLRLMBins |
| GLSZMBins |
| GLSZMDistance |
| MaxPixelValue |
| MinPixelValue |
| NGTDMBins |
| NGTDMDistance |
| NormalizeIntensity |
| VoxelArrayShift |
|  |
| # TextureFeatures |
| Texture_FirstOrder_Energy |
| Texture_FirstOrder_ExcessKurtosis |
| Texture_FirstOrder_InterquartileRange |
| Texture_FirstOrder_MAD |
| Texture_FirstOrder_Max |
| Texture_FirstOrder_Mean |
| Texture_FirstOrder_Min |
| Texture_FirstOrder_RMS |
| Texture_FirstOrder_Range |
| Texture_FirstOrder_Skewness |
| Texture_FirstOrder_Std |
| Texture_FirstOrder_TotalEnergy |
| Texture_FirstOrder_Variance |
| Texture_FirstOrder_rMAD |
| Texture_GLCM_ASM |
| Texture_GLCM_Autocor |
| Texture_GLCM_CP |
| Texture_GLCM_CS |
| Texture_GLCM_CT |
| Texture_GLCM_Contrast |
| Texture_GLCM_Correlation |
| Texture_GLCM_DiffAverage |
| Texture_GLCM_DiffEntropy |
| Texture_GLCM_DiffVariance |
| Texture_GLCM_Entropy |
| Texture_GLCM_Homogeneity |
| Texture_GLCM_HomogeneityNormalized |
| Texture_GLCM_IDM |
| Texture_GLCM_IDMN |
| Texture_GLCM_IMC1 |
| Texture_GLCM_IMC2 |
| Texture_GLCM_InverseVariance |
| Texture_GLCM_MCC |
| Texture_GLCM_MaxProb |
| Texture_GLCM_SumAverage |
| Texture_GLCM_SumEntropy |
| Texture_GLDM_DE |
| Texture_GLDM_DN |
| Texture_GLDM_DNN |
| Texture_GLDM_DV |
| Texture_GLDM_GLN |
| Texture_GLDM_GLV |
| Texture_GLDM_HGLE |
| Texture_GLDM_LDE |
| Texture_GLDM_LDHGLE |
| Texture_GLDM_LDLGLE |
| Texture_GLDM_LGLE |
| Texture_GLDM_SDE |
| Texture_GLDM_SDHGLE |
| Texture_GLDM_SDLGLE |
| Texture_GLRLM_GLV |
| Texture_GLRLM_GNUN |
| Texture_GLRLM_HGRE |
| Texture_GLRLM_LGRE |
| Texture_GLRLM_LRE |
| Texture_GLRLM_LRHGE |
| Texture_GLRLM_LRLGE |
| Texture_GLRLM_RE |
| Texture_GLRLM_RLNUN |
| Texture_GLRLM_RP |
| Texture_GLRLM_RV |
| Texture_GLRLM_SRE |
| Texture_GLRLM_SRHGE |
| Texture_GLRLM_SRLGE |
| Texture_GLSZM_GLN |
| Texture_GLSZM_GLNN |
| Texture_GLSZM_GLV |
| Texture_GLSZM_HGLZE |
| Texture_GLSZM_LAE |
| Texture_GLSZM_LAHGLE |
| Texture_GLSZM_LALGLE |
| Texture_GLSZM_LGLZE |
| Texture_GLSZM_SAE |
| Texture_GLSZM_SAHGLE |
| Texture_GLSZM_SALGLE |
| Texture_GLSZM_SZN |
| Texture_GLSZM_SZNN |
| Texture_GLSZM_ZE |
| Texture_GLSZM_ZP |
| Texture_GLSZM_ZV |
| Texture_Grad_Mean |
| Texture_Grad_Std |
| Texture_Histo_Energy |
| Texture_Histo_Entropy |
| Texture_Histo_ExcessKurtosis |
| Texture_Histo_Max |
| Texture_Histo_Mean |
| Texture_Histo_Min |
| Texture_Histo_Skewness |
| Texture_Histo_Std |
| Texture_Histo_VoxelCount |
| Texture_Moment_J1 |
| Texture_Moment_J2 |
| Texture_Moment_J3 |
| Texture_NGTDM_Busyness |
| Texture_NGTDM_Coarseness |
| Texture_NGTDM_Complexity |
| Texture_NGTDM_Contrast |
| Texture_NGTDM_Strength |
| Texture_Percentile_10 |
| Texture_Percentile_25 |
| Texture_Percentile_50 |
| Texture_Percentile_75 |
| Texture_Percentile_90 |
| Texture_Percentile_95 |
|  |
