# Supplementary material for: Radiomics Is Effective for Distinguishing Coronavirus Disease 2019 Pneumonia From Influenza Virus Pneumonia
Source: Front Public Health. 2021 Jun 15;9:663965. doi: 10.3389/fpubh.2021.663965 (PMC8239147; doi:10.3389/fpubh.2021.663965)
Supplement: Supplementary file 2 [file Data_Sheet_2.docx]

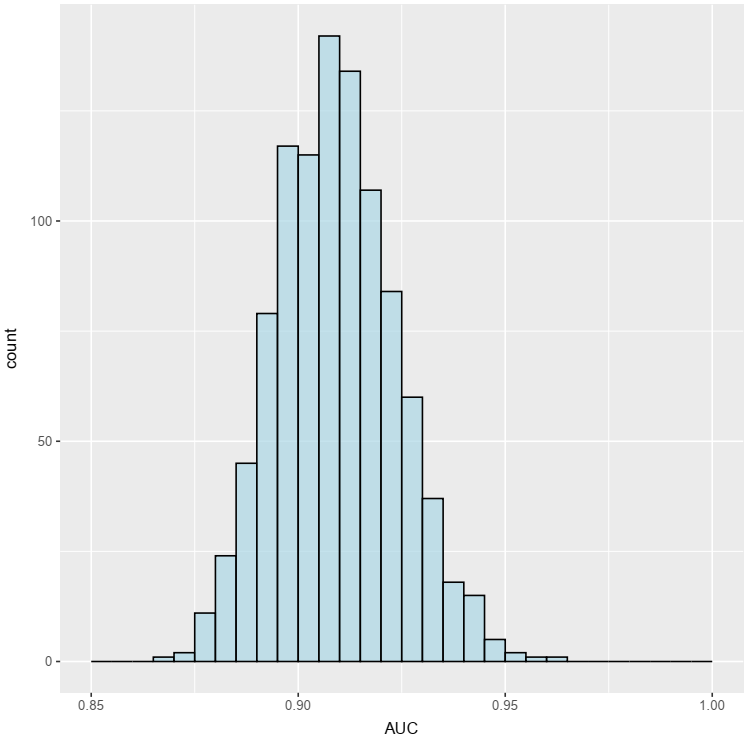


Figure S1. Histograms regarding the distributions of AUCs from the bootstrap method for the radiomics model
